# A Review of Processes for Removing Antibiotics from Breeding Wastewater

**DOI:** 10.3390/ijerph18094909

**Published:** 2021-05-05

**Authors:** Airu Huang, Muting Yan, Jingjun Lin, Lijie Xu, He Gong, Han Gong

**Affiliations:** 1 Joint Laboratory of Guangdong Province and Hong Kong Region on Marine Bioresource Conservation and Exploitation, College of Marine Sciences, South China Agricultural University, Guangzhou 510000, China; Huang-@outlook.com (A.H.); JingJun_Lin@outlook.com (J.L.); 2 College of Biology and the Environment, Nanjing Forestry University, Nanjing 210037, China; xulijie@njfu.edu.cn; 3 School of Chemical Engineering, ShengLi College, China University of Petroleum, Dongying 257000, China; ghstef@163.com

**Keywords:** breeding wastewater, antibiotics, constructed wetland, biological treatment method, advanced oxidation process, membrane technology

## Abstract

Antibiotic pollution has become an increasingly serious issue due to the extensive application of antibiotics, their resistance to removal, and the harmful effects on aquatic environments and humans. Breeding wastewater is one of the most important sources of antibiotics in the aquatic environment because of the undeveloped treatment systems in breeding farms. It is imperative to establish an effective antibiotic removal process for breeding wastewater. This paper reviews the treatment methods used to remove antibiotics from breeding wastewater. The mechanisms and removal efficiency of constructed wetlands, biological treatments, advanced oxidation processes (AOPs), membrane technology, and combined treatments are explained in detail, and the advantages and disadvantages of the various treatment methods are compared and analyzed. Constructed wetlands have high removal rates for sulfonamide (SM), tetracycline (TC), and quinolone (QN). The antibiotic removal efficiency of biological treatment methods is affected by various processes and environmental factors, whereas AOPs and combined treatment methods have better antibiotic removal effects. Although it has broad application prospects, the application of membrane technology for the treatment of antibiotics in breeding wastewater needs further research.

## 1. Introduction

Antibiotics are synthesized with the aim to kill or inhibit other microorganisms and their derivatives. Because antibiotics can inhibit bacterial infection and promote animal growth, they are widely used in the livestock and aquaculture industries [1,2]. The global annual consumption of antibiotics exceeds 100,000 tons [3]. According to estimates by the World Health Organization (WHO), the antibiotics used in the animal husbandry industry account for almost 80% of all antibiotics of major medical significance in some countries. As one of the world’s largest antibiotic producers and consumers, China consumed 92,700 tons of antibiotics in 2013, with 52% of this total used in the animal husbandry industry [4].

The main antibiotics used in the breeding industry in China are chloramphenicol (broad-spectrum antibiotic), tetracycline (inhibits Gram-negative and -positive bacteria), macrolides or MACs (inhibit Gram-positive bacteria), sulfonamide or SM (treatment of furunculosis and vibriosis), and nitrofurans (inhibit and kill bacteria and protozoa). Because some farmers have used antibiotics in large quantities, serious environmental pollution has occurred alongside their economic benefits. Most antibiotics cannot be fully metabolized by animals, and large amounts are excreted into the environment in their original form. Various processes were developed to remove antibiotics in urban water/wastewater treatment plants [5,6]. The treatment of breeding wastewater is less effective than that of urban water systems, and antibiotics cannot be completely removed, resulting in a large amount of antibiotics being discharged into the soil and water bodies. Antibiotics have a very low degradation rate in the natural environment and can persist for long periods. They have been detected in surface water, groundwater, and sediments on all continents [3,7,8]. Antibiotics in water bodies can induce microorganisms to develop resistance [9], and their influence is transmitted to other organisms through the food chain, thereby disrupting the ecological balance. Because of antibiotic pollution in the aquatic environment, the spread and transfer of antibiotic-resistant bacteria (ARB) and antibiotic-resistant genes (ARGs) has become a major public health issue [10]. The WHO defines antimicrobial resistance as one of the three major threats to human health [11].

With the development of the breeding industry and the widespread application of antibiotics, a large amount of wastewater containing antibiotics is produced. If this is discharged into the environment without effective treatment, it will cause serious pollution, altering the ecological balance and endangering human health. Therefore, the removal of antibiotics from breeding wastewater is an important issue. This paper reviews the existing treatment methods for removing antibiotics from breeding wastewater. The mechanism, removal efficiency, and advantages and disadvantages of each removal process are evaluated, providing a basis for further studies of antibiotic pollution remediation in breeding water.

## 2. Treatment Technologies

### 2.1. Constructed Wetlands

A constructed wetland wastewater treatment system is an artificial sewage treatment ecosystem, which uses the combined action of soil, plants, and microorganisms to treat the wastewater that enters the wetland. The wastewater is purified by filtration, adsorption, co-precipitation, ion exchange, plant adsorption, and microbial decomposition [12]. Constructed wetlands have broad-spectrum decontamination capabilities, and are economical, efficient, and easy to maintain and manage [13]. Studies have shown that constructed wetlands remove SM, TC, and quinolone (QN) from breeding wastewater at high rates, which range from 49.43%–85%, to 69%–100%, and 82%–100%, respectively [14,15,16,17]. In addition, constructed wetlands have also been shown to be efficient at removing enrofloxacin (ENR), oxytetracycline (OTC), and ARB from marine aquaculture wastewater [18].

Constructed wetlands can be classified as free water surface flow constructed wetlands (FWS CWs), horizontal subsurface flow constructed wetlands (HSF CWs), and vertical subsurface flow constructed wetlands (VSF CWs) according to the direction of water flow. In vertical subsurface flow wetlands, aerobic and anaerobic activities coexist, and they have a long hydraulic retention time (HRT), which can enhance the degradation of antibiotics by microorganisms [19]. Therefore, the VSF CW is widely used to treat breeding wastewater. Table 1 summarizes the antibiotic removal rates achieved by constructed wetlands.

The performance of constructed wetlands is mainly affected by plant species, fill material type, water depth, hydraulic loading rate (HLR), HRT [23,24,25] and environmental factors [26]. Plants can directly absorb antibiotics, provide attachment points for microorganisms, and increase microbial activity, which can effectively improve the removal of antibiotics from wastewater. For example, the removal rate of sulfadimethoxine by *Azolla* is 56%–86% (initial concentration, 50–450 mg/L) [27]. The removal rate of sulfadiazine, sulfamethazine (SMZ), and sulfamethoxazole (SMX) by ryegrass is 89%–99% (initial concentration, 10–100 μg/L) [20]. The TC removal rate by *Eichhornia crassipes* is higher than 70% (initial concentration, 3.0–15.0 mg/L) [28].

The fill material functions in interception and adsorption, which can enhance the degradation of antibiotics by microorganisms in the fill material [29]. The pore size, specific surface area, and chemical composition of the fill material likely affect the antibiotic removal efficiency. Liu et al. showed that the removal rates of SMZ, OTC, and ciprofloxacin (CIP) in swine wastewater (73%, 95%, and 85%, respectively) using a zeolite-medium vertical flow constructed wetland were slightly higher than when using a volcanic rock-medium vertical flow constructed wetland (68%, 91%, and 82%, respectively) [16]. This may be due to the pH and average pore size of the fill material affecting the removal rate.

The HLR refers to the amount of wastewater per unit volume or unit area that a constructed wetland can treat per day. The greater the HLR, the shorter the HRT [30]. The daily input of antibiotics into a constructed wetland is obtained by multiplying the HLR and the initial antibiotic concentration. Therefore, the HLR also affects the antibiotic removal rate. The removal efficiency for the various antibiotics differs with changes in the HRT. Liu et al. showed that in constructed wetlands with different configurations for removing antibiotics in breeding wastewater, the removal rate of SMX can increase significantly when the HRT is extended from 1 to 3 d [21]. The SMX removal rate in horizontal subsurface flow constructed wetlands (HSF CWs) and integrated vertical flow constructed wetlands (IVF CWs) increased from 4% and 3%, to 59% and 55%, respectively. However, the HRT has been shown to have no significant effects on the removal of ENR and florfenicol. This is because the different structures of these three antibiotics require different removal mechanisms. The SMX removal mechanism is mainly degradation by microorganisms. The ENR removal mechanism is mainly adsorption by the fill material, whereas florfenicol is relatively stable and is difficult to remove in a conventional constructed wetland [31].

In addition, pH affects antibiotic activity, temperature affects the reproduction of microorganisms, and light affects plant growth; thus, these factors all indirectly affect antibiotic removal efficiency [32]. Liao et al. showed that with a HLR of 5 cm/d and after operating for 60 d, the TC, SM, and QN removal rates (72%, 47%, and 22%, respectively) in a celery-based aquatic system in summer were significantly higher than those in a spinach-based aquatic system (33%, 20%, and 7%, respectively) [22]. However, there was no significant difference in antibiotic removal efficiency between the two types of aquatic plant filter beds in winter, which was mainly due to the influence of plant characteristics and environmental factors, such as temperature and pH [33]. Moreover, the advanced media utilization [34] and tidal flow operation in the wetland [35] were also studied. Tidal operation played a positive role in removal of chemical oxygen demand (COD) and NH_4_^+^-N [35]. The treatment performance and the lifespan of CWs were increased with the use of biochar under tidal operation [34].

### 2.2. Biological Treatment

The main antibiotic removal processes during biological treatment include sludge adsorption and biodegradation [36]. In general, biological treatments can be classified as aerobic, anaerobic, and combined aerobic and anaerobic methods according to their different oxygen requirements. The main aerobic method is the biological aerated filter system (BAF). The main anaerobic methods are the anaerobic digestion (AD), upflow anaerobic sludge blanket (UASB), anaerobic filter (AF), and anaerobic baffled reactor (ABR) processes. The most common combined aerobic and anaerobic methods are the sequencing batch reactor (SBR) and membrane bioreactor (MBR) processes. The predominant processes currently used to remove antibiotics in breeding wastewater are the BAF, AD, SBR, and MBR processes. Table 2 summarizes the antibiotic removal rates from breeding wastewater by various biological treatment methods.

#### 2.2.1. The BAF System

The BAF system is a new type of sewage treatment process that combines biological contact oxidation and filtration [42]. It consists of three phases including a solid phase to support microbial growth, a liquid phase to submerge the solid material, and a gas phase for air input [43]. It has the advantages of low operating cost, small footprint, and high degree of automation [44].

Because the packing has a strong biofilm adhesion ability and large specific surface area, it promotes the adsorption of sludge; therefore, the antibiotic removal efficiency is rather high. For example, with an HRT of 40–48 h and HLR of 2.8 cm/h, the total antibiotic removal rate with the BAF process can be as high as 89%–91% [37].

#### 2.2.2. The AD Process

AD includes four stages: hydrolysis, acidification, hydrogen production, and acetic acid and methane production [45]. The AD process has more advantages than the traditional activated sludge method for treating swine wastewater, because it does not require additional aeration equipment or energy investment, and less sludge is produced. However, the treated wastewater and sludge residues could still cause harm to the surrounding environment [46]. Under the conditions of 1.38–2.16 kg chemical oxygen demand (COD)/m^3^·d, an operating temperature of 37 ± 1 °C, and an HRT of 16 d, the TC and QN removal rates from swine wastewater by AD have been reported to be 65% and 85%, respectively [38].

#### 2.2.3. The SBR Process

The SBR system consists of one or more aeration reaction tanks, and the sewage enters the tank in batches. The SBR reactor operates in five sequences: influent feeding, anoxic phase, aerobic phase, sludge settling, and effluent discharge [47]. The operation of the SBR process is flexible and saves land and energy, but its running and management are complicated [48]. In a previous study, a lab-scale intermittently aerated sequencing batch reactor was applied for treatment of swine wastewater. With an HRT of 3–5 d and a minimum COD load, the TC removal rate was 88%, of which removal by sludge adsorption and biological degradation accounted for 30% and 58%, respectively. The SM removal rate was approximately 96%, with almost all of the removal occurring due to biodegradation [39].

#### 2.2.4. The MBR Process

An MBR is a type of wastewater biological treatment technology that combines modern membrane separation technology and biological technology. The MBR has the advantages of long sludge retention time, flexible operation, low sludge output, and high nitrification performance, but the disadvantages are its high energy consumption and operating costs [49]. A previous study has shown that MBRs remove SM and TC from swine wastewater with high efficiency (>90%), whereas the QN removal efficiency was lower (<70%) when the HRT was 33–51 h [40].

Improvements in existing technology in terms of combining different processes have also enhanced the removal of antibiotics from breeding wastewater. For example, some researchers have used biofilm MBRs (BF-MBRs) and compared the effectiveness of this technology in removing antibiotics from piggery wastewater with that of a conventional MBR. When the HRT was 5–4, 3–2, and 1 d, the antibiotic removal rates associated with the BF-MBR were 87%, 80%, and 45%, respectively, whereas those associated with the MBR were only 84%, 57%, and 26%, respectively [50].

The main factors affecting the efficiency of biodegradation are HRT, sludge concentration, membrane type, water quality (pH value, temperature), and the property of the antibiotics [51,52,53]. The removal efficiency of antibiotics can be improved by a longer sludge retention time and higher biomass concentration, both of which increase the contact time between the microorganisms and antibiotics [54]. For example, with an HRT of 5 d and a COD/total nitrogen ratio of 2.1, the total antibiotic removal rate associated with an intermittent aeration membrane bioreactor (IAMBR) was 78%–80%. With the HRT shortened to 3 d, the removal rate of total antibiotics was significantly reduced to 4%–53% [41]. Ben et al. found that when the SBR method was used to treat swine wastewater, the concentration of suspended matter and pH of the solution affected the removal efficiency of the activated sludge method [55]. With an increase in the suspended solid concentration, the SMZ removal rate also increases. The pH of the solution affects the form of SMZ and the surface properties of activated sludge, thus affecting the removal efficiency by activated sludge.

These studies have shown that the biological method is selective in the removal of antibiotics, and the removal efficiency is greatly affected by process parameters and environmental parameters. It therefore has certain limitations for removing antibiotics from breeding wastewater. Therefore, improvement of the removal efficiency when biological methods are applied to remove antibiotics needs to be further studied.

### 2.3. Advanced Oxidation Processes (AOPs)

AOPs are oxidation technologies that use strong oxidizing hydroxyl radicals (·OH) produced in reactions as the main oxidant to decompose and mineralize organic pollutants in water [56]. The ·OH oxidizes organic pollutants through the breakage of chemical bonds or reactions such as electron transfer, addition, and substitution, finally decomposing the pollutants into small molecules of organic matter that are easy to degrade, as well as CO_2_ and H_2_O [57]. The most widely used AOPs for treating antibiotics in breeding wastewater include electrochemical oxidation, the ozonation process, and the Fenton process. Table 3 summarizes the removal efficiencies of antibiotics by various AOPs in breeding wastewater.

#### 2.3.1. Electrochemical Oxidation

Electrochemical oxidation is a technology that produces strong oxidants such as ·OH, HO_2_·, and O_2_^−^ to degrade pollutants through electrode reactions [19]. Miyata showed that the degradation efficiency of TC from livestock and poultry livestock wastewater was as high as 99% after electrochemical treatment for 6 h, with Na_2_SO_4_ as the electrolyte and Ti/IrO_2_ as the anode [58]. Antibiotics including sulfadimidine and norfloxacin, as well as NH_4_^+^-N and COD, were completely removed by the flow-through electro-oxidation process [63]. The removal efficiencies of TC, OTC, chlortetracycline (CTC), and olaquindox (OLA) in simulated livestock wastewater were 98%, 91%, 91%, and 99%, respectively, with the voltage of 5 V, pH of 9, aeration for 3 h, and electrolysis for 2 min [59].

Wang et al. also found that coexisting substances in wastewater affect antibiotic removal efficiency [59]. When the citric acid concentration was 0.02 M, the maximum OLA, TCT, and OTC removal rates were achieved (69%, 56%, and 58%), because the addition of citric acid changed the pH of the wastewater. When the acetic acid concentration was 0.175 M, the OLA, OTC, TC, and CTC removal rates peaked (at 71%, 68%, 60%, and 74%, respectively). Excessively high acetic acid concentrations made the wastewater more acidic, which caused the electrode particles to compete in the adsorption of organic matter, leading to a reduction in the antibiotic removal rate. Sodium dodecyl sulfonate (SDS) improved the removal rate of OLA (when the SDS concentration was 0.02 M, the removal rate was 100%), but it had a strong inhibitory effect on the removal efficiency of TC (when the SDS concentration was 0.02 M, the removal rate decreased to 25%). In addition, the removal rate of OTC and CTC decreased with an increase in the concentration of SDS.

#### 2.3.2. Ozonation Process

There are two mechanisms for ozone-based antibiotic degradation: the direct oxidation of ozone and indirect oxidation through the generation of free radicals (Equation (1)) [64]. As an electrophilic reactant, ozone can attack the aromatic rings and unsaturated double bonds of TC, SM, and QN antibiotics [59], and it can therefore effectively remove these antibiotics. Li et al. showed that with an ozone concentration of 7.8 mg/L and treatment for 20 min, the ozonation process could remove TC, SM, and QN antibiotics from piggery wastewater at levels as high as 96%–98% [60].

3O_3_ + H_2_O → 2OH + 4O_2_ (1)

In addition, the combined processes of ozone oxidation with ultraviolet light (UV), hydrogen peroxide (H_2_O_2_), or catalysts can generate large amounts of ·OH (Equations (2)–(6)), and then degrade organic pollutants [57]. Lee et al. showed that the removal rate of CTC in livestock wastewater was significantly improved from 30% to 65% by the ozone/H_2_O_2_ coupling system compared with ozone oxidation alone [65].

O_3_ + H_2_O_2_ → OH^•^ + O_2_ + HO_2_ (2)

H_2_O_2_ → HO_2_^−^ + H^+^ (3)

H_2_O_2_ → HO_2_^−^ + H^+^ (4)

O_3_ + O_2_^−^ → O_3_^−^ + O_2_ (5)

O_3_^−^ + H_2_O → OH^•^ + OH^−^ + O_2_ (6)

#### 2.3.3. Fenton Process

In the Fenton process, the reagents, i.e., H_2_O_2_ and Fe^2+^, react with each other to generate ·OH radicals (Equation (7)), which then oxidize and decompose antibiotics [66]. Microwave irradiation was employed to enhance the degradation of antibiotics from biogas slurry by microwave-assisted Fenton oxidation [61]. The OTC, TC, CTC, and OLA removal rates were 93%, 91%, 88%, and 67% under a H_2_O_2_ concentration of 40 mg/L, Fe^2+^ concentration of 12 mg/L, initial pH of 4, microwave radiation time of 2 min, and microwave radiation power of 445 W.

Fe^2+^ + H_2_O_2_ → Fe^3+^ + OH + OH^−^ (7)

It is easy to understand that the dosages of H_2_O_2_ and Fe^2+^ affect the efficiency. Apart from that, the pH also affects the reaction rate. Lin et al. found that the H_2_O_2_ input, Fe^2+^ concentration, and initial pH of the water all affected antibiotic removal efficiency in a study of microwave-assisted Fenton oxidation of antibiotics in biogas slurry [61]. With an increase of the H_2_O_2_ input, the removal efficiency of OTC, TC, CTC, and OLA increased at first and then levelled off. Studies have shown that an appropriate increase in the H_2_O_2_ concentration contributes to the production of ·OH, thereby improving antibiotic removal efficiency [67]. With an increase in the Fe^2+^ concentration, the ·OH production increased, and therefore, the removal efficiency of the four antibiotics increased. By contrast, an increase in the initial pH of the water sample caused the removal efficiency of the four antibiotics to decrease. This is because the oxidation-reduction potential of Fenton’s reagent is affected by the initial pH.

#### 2.3.4. The UV/H_2_O_2_ Method

In the UV/H_2_O_2_ method, H_2_O_2_ is decomposed to produce ·OH with UV irradiation (Equation (8)) [68]. The UV/H_2_O_2_ process is relatively stable in operation and can degrade most organic pollutants. It produces less solid waste during the treatment process than the alternative methods and is widely used in organic wastewater treatment [69]. The removal efficiency of SM from livestock wastewater could be higher than 95% by UV/H_2_O_2_ treatment process under the conditions of pH 5.0, UV wavelength of 254 nm, and H_2_O_2_ concentration of 7.0 mM [62].

(8) $$H_{2}O_{2}\;\overset{\;hv}{\to }\;2\cdot \mathrm{OH}$$

Li et al. found that pH, H_2_O_2_ concentration, and reaction time affected antibiotic removal efficiency [62]. They also found that the SM removal efficiency was highest when the initial pH was 5.0. The antibiotic removal efficiency increased with the increase of pH ranging from 1.0–5.0, while the removal efficiency of antibiotics decreased with the increase of pH ranging from 5.0–11.0. Under acidic conditions, H_2_O_2_ is easily decomposed into water and oxygen (Equation (9)). Therefore, the ·OH production decreases, which in turn affects antibiotic removal efficiency. Under alkaline conditions, part of the H_2_O_2_ reacts with OH^−^ to form HO_2_^−^ (Equation (10)), which can react with ·OH (Equation (11)). Therefore, the amounts of ·OH and degradation efficiency reduced. The antibiotic removal rate was highest when the H_2_O_2_ concentration was 7.0 mM. An excessive input of H_2_O_2_ reduced the efficiency of antibiotic removal. Because H_2_O_2_ plays a dual role in the oxidative degradation of antibiotics, an appropriate amount of H_2_O_2_ can produce ·OH, whereas excessive H_2_O_2_ will consume ·OH (Equation (12)). The antibiotic removal rate will also be higher with longer reaction times. For example, when the reaction time was extended from 20 to 60 min, the SM removal rate increased from 80% to 95%.

(9) $$2H_{2}O_{2}\;\overset{\;hv}{\to }\;2H_{2}O+O_{2}$$

(10) $$H_{2}O_{2}\;+{{\mathrm{OH}}^{-}}_{\;}\overset{\;hv}{\to }\;{{\mathrm{HO}}_{2}}^{-}+H_{2}O$$

(11) $$\mathrm{OH}+{{{\mathrm{HO}}_{2}}^{-}}_{\;}\overset{\;hv}{\to }\;H_{2}O+\cdot {O_{2}}^{-}\;$$

H_2_O_2_ + OH^•^→ HO_2_^•^ + H_2_O (12)

These studies have shown that AOPs can effectively remove antibiotics from breeding wastewater; especially, that high removal efficiency can be achieved in a short reaction time. The AOPs therefore have good potential in antibiotic treatment in breeding wastewater.

### 2.4. Membrane Technology

With membrane technology, pollutants are intercepted as wastewater passes through small membrane pores. The methods mainly depend on microfiltration, ultrafiltration, nanofiltration, and reverse osmosis [66]. Membrane technology has the advantages of high work efficiency, simple operation, and low cost. The application of membrane technology for the treatment of antibiotics in wastewater treatment plants has rarely been reported, but there are potential applications for the removal of antibiotics from other types of wastewater. For example, when UV/ozone and nanofiltration were combined to treat wastewater in sewage treatment plants, the antibiotic removal rate was 87%; in addition, the dissolved organic carbon (DOC) decreased by 40%, biodegradability increased 4.6-fold, and ecotoxicity decreased by 58% [70]. In addition, membrane technology could also be used for the removal of other pollutants in breeding wastewater. Lan et al. found that nanofiltration and reverse osmosis processes can be used to treat swine wastewater and can effectively remove various ARGs, nitrogen, phosphorus, and other pollutants [71]. Therefore, further studies on the application of membrane technology to remove antibiotics from breeding wastewater were to be conducted.

### 2.5. Other Processes

In addition to applying a single method to treat antibiotics in breeding wastewater, some combined treatments to improve removal efficiency were developed. For example, Ben et al. studied the degradation of antibiotics in swine wastewater using a combined biological–Fenton process. In this method, SBR was used to perform the biological treatment, and then the Fenton process was used for further treatment [72]. After pretreatment by the SBR method, the removal rate for COD, suspended solids, and total nitrogen in swine wastewater was as high as 95%, which provided favorable conditions for the Fenton process, and then the final removal rates of MACs and SM were as high as 99% and 92%–97%, respectively. Therefore, the integration of an AOP and a biological method can effectively remove antibiotics from breeding wastewater, as well as some conventional pollutants. Qian et al. conducted a study in which swine wastewater was pretreated using an upflow anaerobic sludge layer and SBR process, and then the wastewater was treated with the Fenton process to remove antibiotics [73]. The average antibiotic removal efficiency was 74%. Moreover, Han et al. showed that the antibiotic removal rate was as high as 92% when the SBR and AD methods were combined to treat swine wastewater [74].

Apart from the above technologies, other promising remediation technologies, e.g., the microalgae technique, were also explored [75]. Sun et al. designed a novel microalgae-bacteria powered biofuel cell (MBBFC). Efficient degradation of antibiotic florfenicol (FLO) with simultaneous nitrogen removal was achieved in the bioelectrochemical process.

## 3. Concluding Remarks and Prospects

The fate and mechanisms of treatment processes for removing antibiotics from breeding wastewater, including constructed wetlands, biological treatments, AOPs, and combined treatments, are reviewed in this paper.

(1) Constructed wetlands can be operated economically and are characterized by a strong decontamination ability, high efficiency, easy maintenance and management, and some level of selectivity in the removal of antibiotics.

(2) The degradation of antibiotics by biological treatments is affected by factors such as process parameters, water quality conditions, and environmental factors.

(3) AOPs and combined treatments show a high removal efficiency of antibiotics. They have broad development and application prospects in the treatment of breeding wastewater.

(4) Membrane technology can be used to effectively remove antibiotics, but it is currently seldom used in the removal of antibiotics from breeding wastewater. The application of membrane technology to remove antibiotics from breeding wastewater remains a viable option.

The development of more efficient, low-cost, and easy-to-operate treatment processes for removing antibiotics from breeding wastewater will be a pressing research topic in the future. Faced with the increasingly serious problem of antibiotic pollution, it is necessary to not only continuously improve wastewater treatment processes but also curb the abuse and excessive discharge of antibiotics at their sources. In China, there are clear regulations on the amount of antibiotics to be used in animal husbandry feed, but there are no clear standards for the levels of antibiotics allowed to be discharged in breeding wastewater. Therefore, it is imperative to issue such standards for breeding wastewater as soon as possible.

## Figures and Tables

**Table 1:** Removal of antibiotics in constructed wetlands.

Processes	Antibiotics	Plant	Fill Material	HRT (d)	HLR (cm/d)	Initial Concentration of Antibiotics (μg·L^−1^)	Removal Efficiency (%)	References
Vertical flow constructed wetlands	SM2	*Hybrid pennisetum*	Zeolite	1.25	3	40	73	[16]
			Volcanic rocks				68	
	OTC		Zeolite				95	
			Volcanic rocks				91	
	CIP		Zeolite				85	
			Volcanic rocks				82	
Artificial plant floating bed system	SD	Ryegrass	-	-	-	100	99	[20]
	SMZ					100	999–100	
	SMX					10	89–92	
Horizontal subsurface flow constructed wetlands	SMX	*Phragmits communis*	Gravel and zeolite	1	25.2	0.0987	4	[21]
				3			59	
Integrated vertical flow constructed wetlands	SMX	*Phragmits communis*	Gravel and zeolite	1	8.4	0.0987	3	[21]
				3			55	
Aquatic plant filter bed	TCs	Celery	-	60	5	30	33	[22]
		Spinach					72	
	SMs	Celery					20	
		Spinach					47	
	QNs	Celery					7	
		Spinach					22	

**Table 2:** Removal of antibiotics from breeding wastewater by biological processes.

Processes	Biological Treatment	Antibiotics	Operation Conditions	Removal Efficiency (%)	References
Aerobic methods	BAF	SMM, SCP, SMZ, TMP, NOR, OTX, LIN, LCM, OTC	HRT = 40–48 h, HLR = 2.8 cm/h	89–91 (total antibiotics)	[37]
Anaerobic methods	AD	TCs	1.38–2.16 kg COD/m^3^·d, 37 ± 1 °C, HRT = 16 d	65	[38]
		QNs		85	
Aerobic–anaerobic combined methods	SBR	TC	HRT = 3–5 d	88	[39]
		SMs		96	
	MBR	SMs	HRT = 33–51 h	>90	[40]
		TCs		>90	
		QNs		<70	
	IAMBR	TC, CTC, OTC, DC, SMX, SMZ, CIP, NOR, ENR, TYL, RTM	COD/TN = 2.1, HRT = 3 d	4–53 (total antibiotics)	[41]
			COD/TN = 2.1, HRT = 5 d	78–80 (total antibiotics)	[41]

Notes: SMM = sulfamonomethoxine, SCP = sulfachloropyridazine, TMP = trimethoprim, NOR = norfloxacin, OFX = ofloxacin, LIN = lincomycin, LCM = leucomycin, TC = tetracycline, CTC = chlortetracycline, DC = doxycycline, TYL = tylosin, RTM = roxithromycin, COD = chemical oxygen demand, TN = total nitrogen.

**Table 3:** Removal of antibiotics from breeding wastewater by advanced oxidation processes.

Processes	Antibiotic	Operation Conditions	Initial Concentration of Antibiotics (mg/L)	Removal Efficiency (%)	References
Electrochemical oxidation	TCs	Na_2_SO_4_ as electrolyte and Ti/IrO_2_ as anode, electrochemical treatment was carried out for 6 h	100	99	[58]
	TC	Voltage = 5 V, pH = 9, aeration time = 3 h, electrolysis = 2 min	2.5	98	[59]
	OTC		2.0	91	
	CTC		2.0	91	
	OLA		2.0	99	
Ozonation process	TCs	[O_3_]_0_ = 7.8 mg/L, t = 20 min	(5.846–8.444) × 10^−3^	96	[60]
	SMs		(1.395–3.341) × 10^−3^	98	
	QNs		(3.709–4.915) × 10^−3^	97–98	
Fenton process	OTC	[H_2_O_2_]_0_ = 40 mg/L, [Fe^2+^]_0_ = 12 mg/L, pH_0_ = 4, microwave radiation time = 2 min, microwave radiation power = 445 W	1.3	93	[61]
	TC		1.3	91	
	CTC		1.8	88	
	OLA		2.8	67	
UV/H_2_O_2_ process	SMs	pH = 5.0, UV = 254 nm, [H_2_O_2_]_0_ = 7.0 mM	2.0	95	[62]

## Data Availability

No new data were created or analyzed in this study. Data sharing is not applicable to this article.
